# IVIVC from Long Acting Olanzapine Microspheres

**DOI:** 10.1155/2014/407065

**Published:** 2014-01-22

**Authors:** Susan D'Souza, Jabar A. Faraj, Stefano Giovagnoli, Patrick P. DeLuca

**Affiliations:** ^1^Sunovion Pharmaceuticals Inc, Marlborough, MA 01752, USA; ^2^Fresenius Kabi USA, Skokie, IL 60077, USA; ^3^Department of Chemistry and Technology of Drugs, Università degli Studi di Perugia, Via del Liceo 1, 06123 Perugia, Italy; ^4^University of Kentucky College of Pharmacy, Lexington, KY 40536, USA

## Abstract

In this study, four PLGA microsphere formulations of Olanzapine were characterized on the basis of their *in vitro* behavior at 37°C, using a dialysis based method, with the goal of obtaining an IVIVC. *In vivo* profiles were determined by deconvolution (Nelson-Wagner method) and using fractional AUC. The *in vitro* and *in vivo* release profiles exhibited the same rank order of drug release. Further, *in vivo* profiles obtained with both approaches were nearly superimposable, suggesting that fractional AUC could be used as an alternative to the Nelson-Wagner method. A comparison of drug release profiles for the four formulations revealed that the *in vitro* profile lagged slightly behind *in vivo* release, but the results were not statistically significant (*P* < 0.0001). Using the four formulations that exhibited different release rates, a Level A IVIVC was established using the deconvolution and fractional AUC approaches. A nearly 1 : 1 correlation (*R* ^2^ > 0.96) between *in vitro* release and *in vivo* measurements confirmed the excellent relationship between *in vitro* drug release and the amount of drug absorbed *in vivo*. The results of this study suggest that proper selection of an *in vitro* method will greatly aid in establishing a Level A IVIVC for long acting injectables.

## 1. Introduction

Establishing an IVIVC (*in vitro in vivo* correlation) remains a challenge for non-oral dosage forms like long acting injectables. One reason for the lack of IVIVC is the dearth of *in vitro* methods that are simple to set up and use, while suitably mimicking *in vivo* conditions. The benefits of establishing an *in vitro in vivo* correlation (IVIVC) have been enumerated in numerous pharmaceutical publications spanning the last three decades. Indeed, IVIVC has remained a topic of constant discussion with several dosage forms, especially solid orals, since the publication of an IVIVC guidance by the FDA in 1997 [1]. The goal of an IVIVC is to establish a relationship between the *in vitro* dissolution behavior and *in vivo* performance of a drug product. An IVIVC is generally described by a linear relationship between parameters derived from the *in vitro* and *in vivo* experiments as quantified by the Pearson correlation. As defined by the FDA guidance, these correlations have been classified under four categories.

- Level A, the highest correlation, is a point to point correlation between *in vitro* dissolution and *in vivo* absorption over time. With a Level A correlation, the *in vitro* dissolution profiles are generally superimposable with *in vivo* absorption curves or may be made superimposable by use of an appropriate scaling factor.
- Level B is a correlation between summary parameters such as *in vitro* dissolution rate and *in vivo* absorption rate (e.g., mean dissolution time (MDT) versus mean residence time (MRT)). Though frequently used, a Level B correlation is not a point to point correlation as it does not uniquely reflect the actual *in vivo* profile due to the fact that several *in vivo* curves will produce a similar MRT value or mean *in vitro* dissolution curve.
- Level C describes a single point comparison of the amount dissolved *in vitro* at a particular time (e.g., *T*_50_%) and an *in vivo* pharmacokinetic parameter (e.g., area under the curve (AUC)). Thus, a Level C correlation is not descriptive of the complete shape of the *in vivo* release profile, which is an important aspect in the characterization of performance from extended release drug products.
- Level D is a rank order correlation that is qualitative in nature.

Once established, an IVIVC can be used to guide formulation and/or process development changes in the various stages of drug development and also simplify any scale-up or post-approval changes.

Additionally, an IVIVC allows setting of clinically relevant *in vitro* dissolution specifications to ensure product quality. A particular benefit of an IVIVC is that it can be used to support the use of dissolution testing as a surrogate for human bioequivalence studies, which would reduce the number of human studies needed for drug applications. A well-described *in vitro in vivo* relationship could also be used to set clinically meaningful dissolution specifications for monitoring drug manufacture. Though the FDA guidance provides a framework for developing and establishing an IVIVC, several challenges have been noted with solid oral dosage forms, particularly, the development of an *in vitro* dissolution method. Among other variables, including the fact that the gastrointestinal environment is highly complex, developing an *in vitro* dissolution method to mimic *in vivo* conditions is not simple or straightforward.

Development of an IVIVC with non-oral dosage forms is even more complex. With the increasing number of such dosage forms being developed and commercialized in recent years, development of an IVIVC has gained additional significance. As such, the FDA guidance is currently applicable only to oral extended release products and not for non-oral dosage forms like transdermals or long acting injectables. However, a few publications have attempted to establish an IVIVC from non-oral dosage forms using the fundamental principles in the guidance [2–4].

Some of the most successful non-oral dosage forms marketed over the past decade include polymeric based injectable dosage forms formulated using polylactide-co-glycolide (PLGA) polymers [6, 7]. These polymers are FDA approved for surgical sutures and implantable devices and have excellent safety, tolerability, and toxicity [8, 9]. Further, they are known to be biodegradable and biocompatible as they undergo biodegradation over weeks to months resulting in the formation of lactic and glycolic acids that are cleared by the Krebs cycle. Several reports have documented the incorporation of a wide range of complex therapeutics like peptides and proteins into PLGA to form micron sized dosage forms called polymeric microspheres [10–14]. Key advantages of delivering therapeutics using polymeric microspheres include sustained drug release, reduced dosage, and fewer systemic side effects. Additional benefits of using polymeric microspheres are the improvement in patient compliance to drug therapy, primarily due to reduced frequency of administration of the slow degrading dosage form that releases the therapeutic in a sustained fashion [15–19]. For this reason, long acting microsphere dosage forms of several molecules like Risperidone (atypical antipsychotic to treat schizophrenia) and Leuprolide (LHRH superagonist against prostate cancer) have been developed and have achieved significant commercial success [20–23].

Despite the advances in development of long acting injectables, literature on the IVIVC with these dosage forms continues to remain sparse. A major challenge cited for the lack of IVIVC is the absence of a standardized or compendial method to assess *in vitro* drug release from long acting injectables [7]. A few authors have attempted Level A, B, and C correlations, albeit with different methods and varying degrees of success [2, 4, 24]. Of the methods available to assess *in vitro* drug release, a dialysis based method offers the most advantages in terms of simplicity in set-up, ease of sampling, and reproducibility. With dialysis based techniques, the dosage forms are entrapped inside a dialysis bag containing media (inner media, non-sink conditions). This dialysis bag is subsequently immersed inside a larger vessel containing a large volume of the same media (outer media, sink conditions), thus enabling a physical separation of the dosage form from the outer media. As drug is released from the dosage form and into the inner media, it diffuses through the dialysis membrane into the outer sink. This scenario mimics *in vivo *conditions where the long acting injectable is immobilized upon subcutaneous or intramuscular administration and surrounded by a stagnant layer leading to slow drug diffusion due to non-maintenance of sink conditions [25, 26]. Several adaptations of the dialysis based techniques (e.g., dialysis bags) have been evaluated, including the modified dialysis method that was successfully used to study drug release from large molecules like peptides [27].

In a previous publication by the same group, long acting injectable formulations of Olanzapine (a second generation atypical antipsychotic) were prepared using four PLGA polymers with the aim of improving effectiveness and patient compliance for this drug. Subsequently, these formulations were administered to rats to assess *in vivo* performance and were successfully shown to provide sustained *in vivo* levels of Olanzapine for 7 to 15 days [5]. In the current study, the Olanzapine PLGA formulations were further characterized for *in vitro* release behavior, using a modified dialysis method, with the goal of achieving an IVIVC.

## 2. Materials and Methods

### 2.1. Materials

Olanzapine was purchased from Cipla Ltd., Bombay, India. PLGA having molecular weights 15, 30, 82, and 131 kDa was purchased from Boehringer Ingelheim (Ingelheim, Germany) and Alkermes (Cambridge, MA, USA). Spectra/Por Dialysis membranes (MWCO 300,000 Da) were purchased from Spectrum Labs, Inc., CA. All the other chemicals were obtained commercially as analytical grade reagents.

### 2.2. Preparation of Microspheres

Olanzapine PLGA microspheres were prepared by a solvent extraction/evaporation method and recovered by filtration [5]. Briefly, a solution of drug and polymer was injected into an aqueous continuous phase under stirring with a Silverson L4R mixer (Silverson machines, MA, USA) at predetermined speeds. Subsequently, the solvents were removed by stirring after which the microspheres were recovered by filtration, suspended in a suitable vehicle, filled into vials, and freeze dried. Briefly, the four formulations prepared were as follows:

- Formulation A (15 kDa, 75 : 25 lactide : glycolide),
- Formulation B (30 kDa, 50 : 50 lactide : glycolide),
- Formulation C (82 kDa, 65 : 35 lactide : glycolide),
- Formulation D (131 kDa, 75 : 25 lactide : glycolide).

### 2.3. Drug Content

Olanzapine content in the microspheres was analyzed by a reverse phase HPLC method using a HPLC C-18 column at a flow rate of 1.5 mL/min. in a gradient mode. The mobile phases were 0.1% TFA (trifluoroacetic acid) aqueous solution and Acetonitrile with 0.1% TFA. Measurements were performed in triplicate. Drug content (%) was expressed as the “weight of drug in microspheres/weight of microspheres × 100” and was determined to be 26, 30, 40, and 40% for Formulations A, B, C, and D, respectively [5].

### 2.4. *In Vitro* Release

*In vitro* release (*n* = 3) was performed using a modified dialysis method [26]. Briefly, Olanzapine microspheres were accurately weighed and placed in a 7 mL dialysis tube (Tube-O-Dilalyzer, MWCO 300,000 Da) filled with 5 mL 0.5 M PBS (phosphate buffered saline), pH 7.4, containing 0.05% Tween 80 and 0.1% sodium azide (inner media), which in turn was placed in a 50 mL tube containing 40 mL of the same release medium (outer media). The contents of the outer media were continuously stirred with a magnetic stirrer to prevent formation of an unstirred water layer at the outer dialyzing surface. At predetermined intervals, 1.0 mL samples were withdrawn from the outer media followed by buffer replacement and HPLC analysis.

### 2.5. *In Vivo* Study

The *in vivo* release of Olanzapine from PLGA microspheres has been described previously [5]. Briefly, male Sprague-Dawley rats (*n* = 6) weighing approximately 300 g were used to evaluate *in vivo* performance of Olanzapine microspheres. The microspheres were injected subcutaneously at the back of the neck at a 10 mg/kg dose (Formulations A and B) or 20 mg/kg dose (Formulations C and D) after reconstitution with water for injection. Blood samples were collected from the tail vein at specific time points. The samples were centrifuged in Microtainer tubes (Becton Dickinson, Franklin Lakes, NJ) and serum was collected. Serum samples were frozen and stored at −20°C until analysis. Serum samples were analyzed at Medtox Laboratories location using a validated method.

### 2.6. IVIVC

The relationship between % drug released *in vitro* and the percent absorbed for the four Olanzapine PLGA formulations was assessed using two approaches.

(a) Nelson-Wagner approach: the fraction absorbed (*F*_abs_) was determined from the plasma concentration-time data by deconvolution using the Nelson-Wagner method as described in [28],
  (1) $$\begin{array}{c} F_{\text{abs}}\left(t\right)=\frac{\left[C\left(t\right)+k_{e}\times {\text{AUC}}_{\left(0-t\right)}\right]}{\left[k_{e}\times {\text{AUC}}_{\left(0-\inf\right)}\right]}. \end{array}$$
 With the Nelson-Wagner equation, the pharmacokinetic profile is deconvoluted to obtain the *in vivo* absorption as a function of time and is plotted alongside the *in vitro* release data to assess the superimposability of the two profiles. If the two curves are superimposable and a linear relationship is obtained, it suggests a strong correlation between *in vivo* and *in vitro* drug release.
(b) Fractional AUC approach: the area under the curve (AUC) was calculated using the trapezoidal rule
  (2) $$\begin{array}{c} \text{AUC}\left(t_{1}-t_{2}\right)=\left[\frac{\left(C_{1}+C_{2}\right)}{2}\right]\times \left(t_{2}-t_{1}\right). \end{array}$$
 The fractional AUC was determined by dividing cumulative AUC at time “*t*” with cumulative AUC_(0−last)_, as described in previous publications [3, 29] and plotted along with the % drug released *in vitro*. In a manner similar to the Nelson-Wagner approach, the superimposability of the *in vivo* and *in vitro* drug release was compared.

## 3. Results and Discussion

### 3.1. *In Vitro* Release

**Figure 1:**
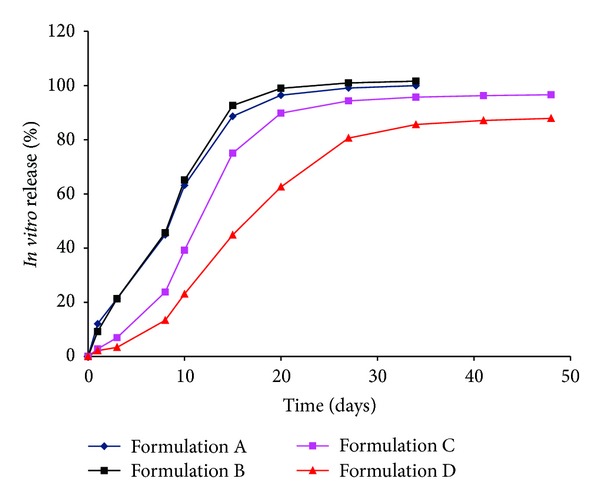
*In vitro* release of Olanzapine PLGA microspheres.

**Figure 2:**
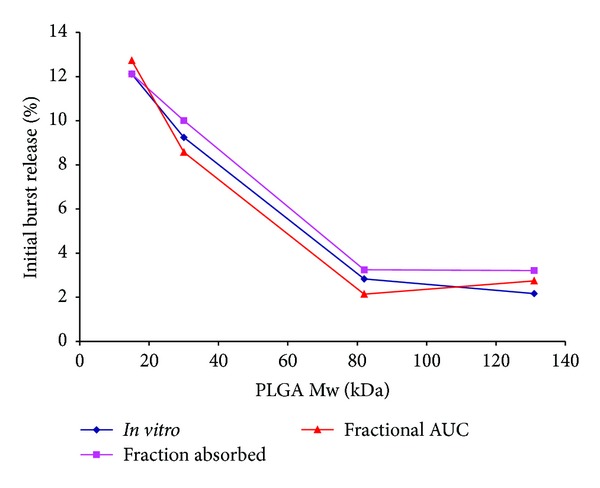
Effect of PLGA molecular weight on initial burst release of Olanzapine.

Figure 1 shows the *in vitro *release from Olanzapine microspheres, measured using the modified dialysis method [26]. An initial burst of almost 10% (day 1) was observed from Formulations A and B after which drug release from these batches was very similar through day 30. In contrast, Formulations C and D exhibited a slight initial burst (day 1) followed by slow release of drug through 3 days (3–7%), by which time nearly 21% of drug release had occurred from Formulations A and B. Interestingly, linear inverse relationship and an excellent correlation (*R* ^2^ > 0.99) were observed between initial burst release *in vitro* and polymer molecular weight for Formulations A–C (15, 30, and 82 kDa, resp.) with a plateau noted at the high molecular weight Formulation D (131 kDa) (Figure 2). From day 3 to 8, Formulation C demonstrated an increase in release rate resulting in 23% drug release while Formulation D lagged with only 13% of drug being released. By day 15, Formulations A and B exhibited more than 90% drug release whereas values for Formulations C and D were around 75% and 44%, respectively. Thus by day 15, drug release from Formulation D was slightly less than half of that observed with that observed with Formulations A and B. This trend continued until complete release was achieved by all four formulations.

Noteworthy observations from the *in vitro* release experiments include:

- All four formulations exhibited initial burst release, the extent of which was governed largely by polymer molecular weight.
- Drug release profiles were sigmoidal or triphasic.
- Rank order of drug release was evident, with Formulations A and B exhibiting rapid release, and Formulation D being the slowest.
- The modified dialysis method was able to suitably capture all phases of the sigmoidal release profile and was discriminatory in nature as it clearly demonstrated rank order of drug release for the formulations investigated.

Overall, the drug release profile from the four formulations can be explained as follows. Once formulated as drug-polymer microspheres, it is well known that release of encapsulated drug from a PLGA matrix is controlled by two phases, namely, drug diffusion through the polymer matrix followed by polymer degradation and erosion [30]. During the first phase, release of the encapsulated drug occurs mainly through its diffusion through the polymer matrix while during the second phase, the release is mediated through both diffusion of the drug and the degradation of the polymer matrix. Drug diffusion through the polymer matrix, a slower process, occurs during polymer hydration. Once hydrated, the polymer undergoes bulk hydrolysis that causes rapid polymer degradation, erosion, and loss of mass. Drug release during the hydrolytic degradation phase (erosional phase) occurs at a much faster pace than that during the diffusional phase.

As such, Formulations A and B, manufactured using low molecular weight PLGA (15 and 30 kDa, resp.), released drug rapidly. Clearly, hydration of the polymeric matrix was extremely rapid (within a day) due to low polymer molecular weight leading to a fast onset of polymer erosion. Despite the differing lactide : glycolide content in the polymers (75 : 25 in Formulation A and 50 : 50 in Formulation B), *in vitro* drug release profiles were nearly superimposable for these two formulations, indicating that lower molecular weight was a major determinant of drug release rate. For the higher molecular weight PLGA (82 and 131 kDa for Formulations C and D, resp.), a classic triphasic release profile was observed; initial burst by day 1 was followed by diffusional release through day 3 and subsequently, erosional release. A faster release rate with Formulation C was not totally unexpected due to a combination of an intermediate polymer molecular weight in a 65 : 35 copolymer. On the other hand, a high polymer molecular weight (131 kDa) in a slow degrading copolymer (75 : 25 lactide : glycolide) led to slow release from Formulation D. Obviously, the rate and extent of *in vitro* drug release depended chiefly on polymer molecular weight than the lactide : glycolide ratio of the PLGA copolymer.

### 3.2. *In Vivo* Results

#### 3.2.1. Fraction Absorbed

**Figure 3:**
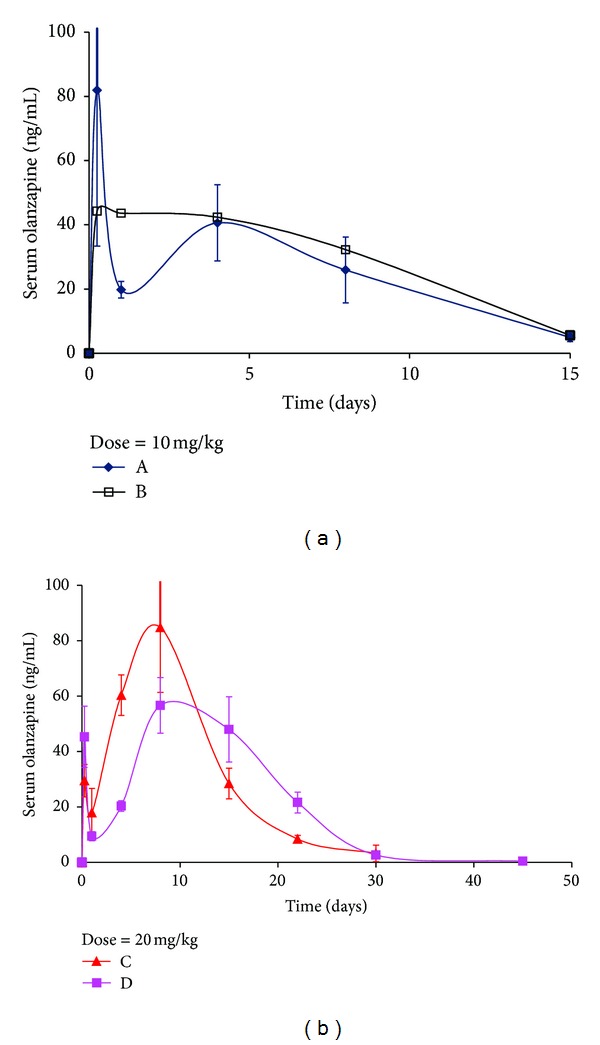
*In vivo* release of Olanzapine from PLGA microspheres (Formulations A and B = 10 mg/kg dose and Formulations C and D = 20 mg/kg dose) (from [5]).

Formulations A and B were administered to rats at a 10 mg/kg dose while Formulations C and D were administered at a 20 mg/kg dose as shown in Figure 3 [5]. Using the Nelson-Wagner method (described in Section 2.6), the fraction of drug absorbed was calculated by deconvoluting the pharmacokinetic profile to obtain the *in vivo* absorption as a function of time [28]. Deconvolution is a numerical method used to estimate the time course of drug input using a mathematical function and is based on a convolution integral. Once deconvoluted, the *in vivo* curve is plotted alongside the *in vitro* release data to assess the superimposability of the two profiles. Thus, the deconvolution approach allows comparison of *in vivo* release profile with *in vitro* behavior.

**Figure 4:**
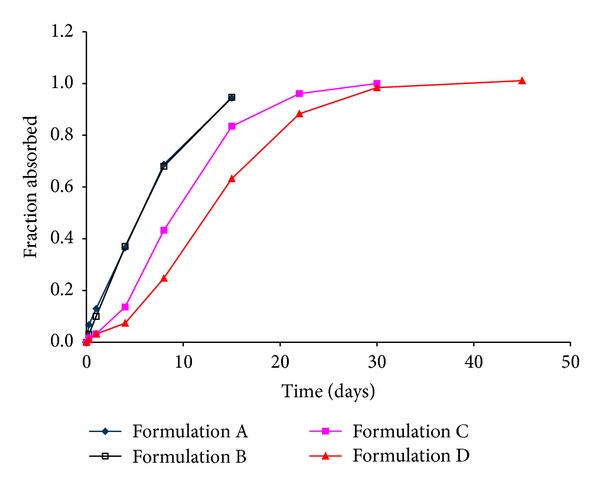
Fraction absorbed *in vivo* (Nelson-Wagner method).

A plot of the fraction of drug absorbed for the four formulations is illustrated in Figure 4. As with Figure 1, the four formulations can be instantly discriminated on the basis of rank order. The profiles for Formulations A and B are nearly identical and reveal that drug absorption from both formulations is rapid. A slower absorption profile is noted with Formulation C with the slowest rate of absorption for Formulation D. Indeed, Figure 4 reveals that nearly 10% of Olanzapine was absorbed by day 1 for the rapidly releasing Formulations A and B, while the remaining formulations released only about 3% at the same time point. In a manner similar to that observed with *in vitro* release, an excellent inverse linear relationship (Figure 2) was observed between initial burst release as measured by the Nelson-Wagner method and polymer molecular weight (*R* ^2^ > 0.99) for Formulations A–C, with levels demonstrating a plateau for the highest molecular weight microsphere formulation (Formulation D). By day 4, only 7% of Olanzapine was absorbed from *Formulation D* whereas the value for Formulation C was almost double that amount. Likewise, the fraction of Olanzapine absorbed from Formulation D was five-fold lower than that of Formulations A and B. This trend continued until complete absorption was achieved for all the formulations, albeit at different time points. A noteworthy fact is that the *in vivo* drug release profiles (Nelson-Wagner method) were triphasic for the four formulations, suggesting a similar mechanism of release (diffusional and erosional) as observed *in vitro*.

#### 3.2.2. Fractional AUC

**Figure 5:**
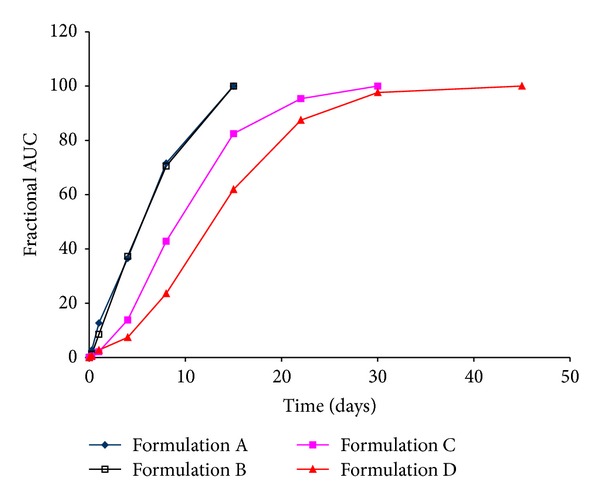
Fractional AUC profile of Olanzapine PLGA microspheres.

A plot of fractional AUC over time is shown in Figure 5 and demonstrates a rank order behavior. Similar to the *in vitro* results (Figure 1), the fractional AUC profiles for Formulations A and B are nearly superimposable and demonstrate that complete release occurs rapidly *in vivo*. On the other hand, a moderate *in vivo* release profile is noted for Formulation C with the slowest *in vivo* profile for Formulation D. As such, the rank order for fractional AUC profile appears similar to the *in vitro* release profile. Of particular note is that fractional AUC values by day 1 are around 11% for Formulations A and B, and between 2 and 3% for Formulations C and D are essentially indistinct from those seen in Figure 1. Akin to the inverse linear relationship observed between initial burst release and polymer molecular weight (Figure 2) for *in vitro* release and fraction absorbed (Nelson-Wagner method), a good correlation was observed with fractional AUC (*R* ^2^ > 0.96). Further, as observed with the *in vitro* release profile (Figure 1), Formulations C and D show slow release through day 3, after which the *in vivo *release rate increases rapidly until approximately 80–85% is released after which the release rate tempers to achieve complete release by days 30 and 45 for Formulations C and D, respectively. Lastly, the *in vivo* fractional AUC plots for the four formulations were sigmoidal in nature, similar to those seen with *in vitro* drug release profiles, reaffirming that the mechanism of release was unchanged whether *in vitro* or *in vivo*.

### 3.3. IVIVC

Per the 1997 FDA guidance, three or more formulations of different release rates are recommended for the purpose of establishing an IVIVC [1]. Additionally, the guidance recommends use of the Nelson-Wagner or Loo-Reigelman method to calculate absorption profile of the drug. Since the four formulations used in the current study had varying release rates *in vivo *and *in vitro*, that is, Formulations A and B had the fastest release rate, while Formulation C was intermediate and Formulation D had the slowest release rate, all four formulations were selected for data analysis and to establish an IVIVC.

Of the FDA recommended methods for IVIVC development, the Nelson-Wagner was deemed suitable for the current study as it is appropriate for use in drugs whose pharmacokinetics can be fitted to one compartment model [28]. Once the fraction absorbed is calculated, a correlation may be obtained by comparing *in vivo* behavior with *in vitro* release, to establish an IVIVC. Another approach suggested by Woo et al. is to compare the fractional AUC with *in vitro* release [29]. In the current study, data analysis was performed using both approaches. Figure 4 shows the fraction of drug absorbed *in vivo*, as determined by the Nelson-Wagner method, while Figure 5 outlines the fractional AUC for the four formulations. At first glance, the similarities between Figures 1, 4, and 5 are very apparent. The release rates for the four formulations follow a rank order where Formulations A and B behave similarly and exhibit fast release while Formulation C demonstrates a modest release rate with the slowest drug release rate from Formulation D.

**Figure 6:**
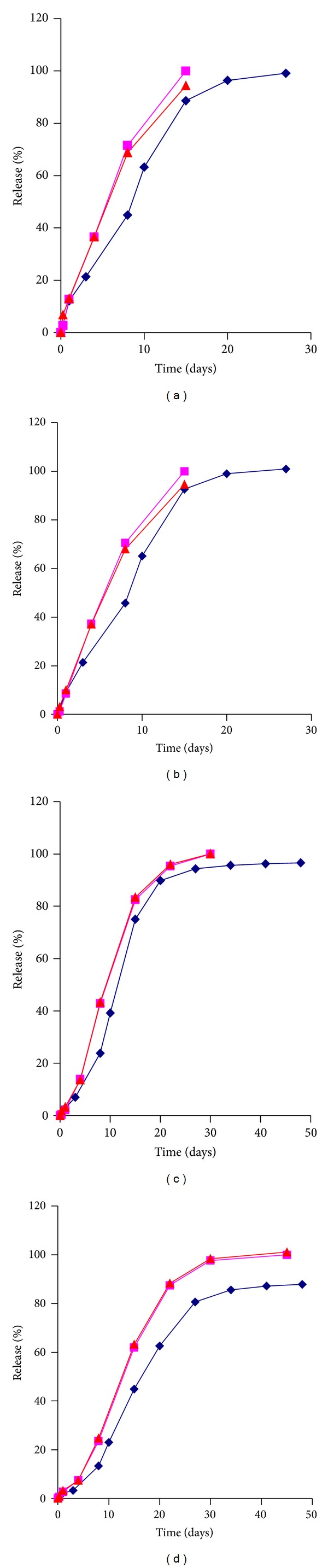
Comparison of *in vitro *and *in vivo *release profiles for Olanzapine microspheres (diamonds = *in vitro* release, squares = fractional AUC, and triangle = Nelson-Wagner absorption).

A comparison between % release *in vitro*, % absorbed using the Nelson-Wagner method, and fractional AUC is depicted in Figure 6. To determine the % absorbed by the Nelson-Wagner method, the fraction absorbed was multiplied by 100. A few striking observations are evident in Figure 6.

- The % release *in vivo* curves, as fractional AUC or using the Nelson-Wagner method, are nearly superimposable for the four formulations. Similar findings were reported by Chu et al. in a study on PLGA microspheres containing the alkaloid, Huperzine A [3].
- *In vitro* release lagged slightly behind *in vivo* release for the four formulations. However, the *in vitro* curves ramp up with a similar slope and are essentially parallel to the *in vivo* profile. In a separate study, Jiang et al. attributed the faster release rate *in vivo* to the contribution of enzymes and foreign body response [31].

**Figure 7:**
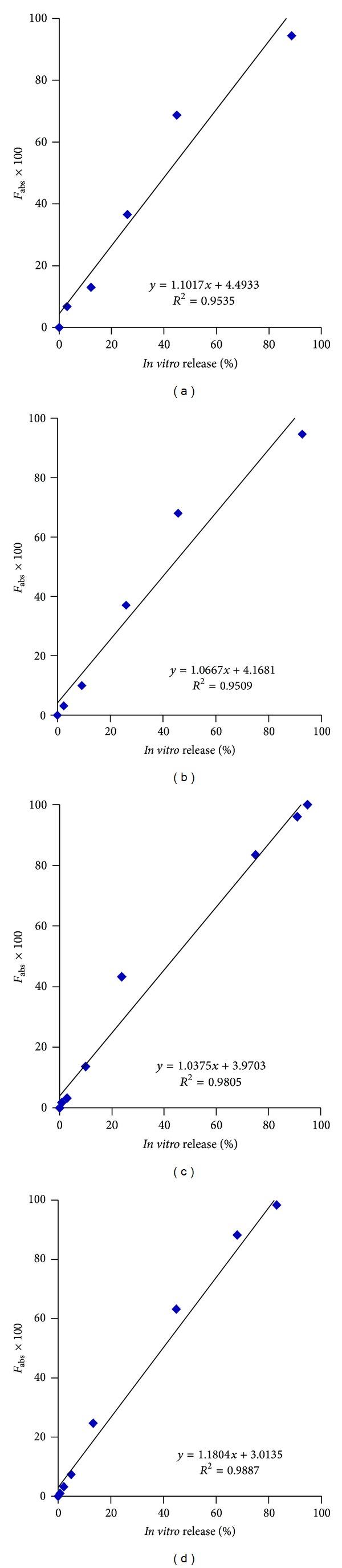
Level A IVIVC for Olanzapine PLGA microspheres using Nelson-Wagner method.

Figure 7 highlights the correlation between % *in vitro* release using the modified dialysis method and the % absorption, as calculated by the Nelson-Wagner method. As can be clearly seen in the figure, an excellent linear correlation (*R* ^2^ values between 0.95 to 0.98, *P* < 0.0001) was obtained for the slow, intermediate and fast releasing formulations. The values of the slope are slightly greater than 1 (1.03–1.18) indicating that *in vivo* release occurred slightly faster than *in vitro* release.

**Figure 8:**
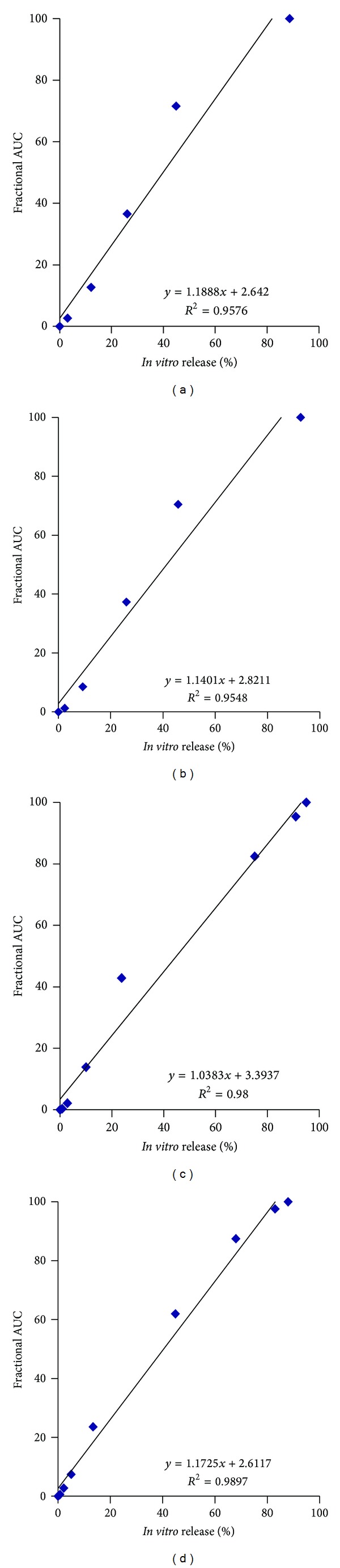
Level A IVIVC for Olanzapine PLGA microspheres using fractional AUC.

An IVIVC utilizing fractional AUC (*in vivo* drug levels) and *in vitro *drug release over time for the four formulations is shown in Figure 8. Once again, a strong linear correlation was obtained (*R* ^2^ values between 0.95 and 0.98, *P* < 0.0001) for the four formulations. Values of the slope ranged between 1.03 and 1.18, similar to those obtained with the Nelson-Wagner method and indicated that *in vivo* release proceeded slightly faster than *in vitro* release. These data suggest that fractional AUC could be used as an alternative to the deconvolution approach to obtaining an IVIVC, similar to that reported previously [3].

**Figure 9:**
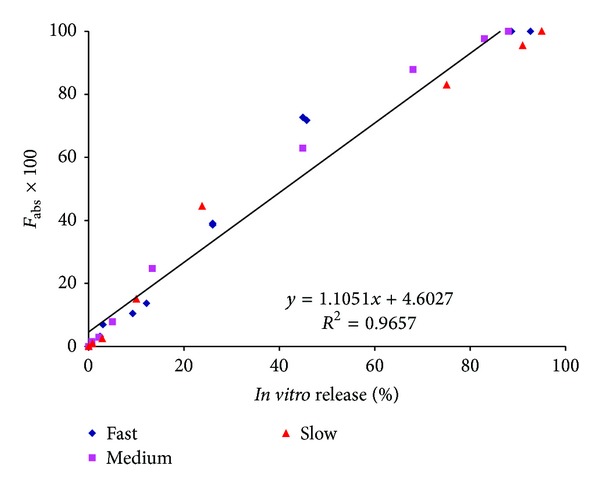
Pooled IVIVC for Olanzapine PLGA microsphere formulations using Nelson-Wagner method (Level A).

**Figure 10:**
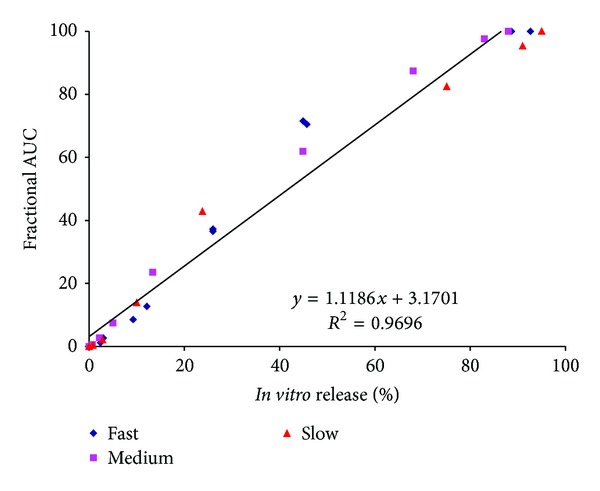
Pooled IVIVC for Olanzapine PLGA microsphere formulations using fractional AUC (Level A).

Figures 9 and 10 highlight the pooled IVIVC for the fast, medium, and slow releasing Olanzapine PLGA formulations. This type of an approach has been recommended by the FDA [1] and has been used by other authors who attempted a Level A correlation with injectable implants having varying release rates [4]. As expected, slopes using the Nelson-Wagner (Figure 9) and fractional AUC (Figure 10) approaches were around 1.10 and 1.12, respectively, once again confirming that *in vivo* release for the fast, medium, and slow formulations followed a similar trend in which there was minimal lag *in vivo*. The results in Figures 9 and 10 demonstrate an excellent correlation (*R* ^2^ value greater than 0.96) between *in vitro *release of Olanzapine from PLGA microspheres and *in vivo* release, represented by fractional AUC and the FDA approved Nelson-Wagner method. To our knowledge, the results in this study are the first time an IVIVC has been attempted and successfully achieved with Olanzapine PLGA microspheres.

Further, these results suggest that assessment of *in vitro* release using the modified dialysis method is an excellent predictor of *in vivo* behavior of a small molecule, Olanzapine, formulated with PLGA of varying molecular weight and copolymer composition. Similar results were reported on peptide PLA and PLGA microspheres. For instance, Woo et al. used the modified dialysis method to evaluate *in vitro* release from Leuprolide PLGA microspheres [29]. In another study, Kostanski et al. used the modified dialysis method with Orntide microspheres formulated with 50 : 50, 75 : 25, and 85 : 15 PLGA and also Orntide PLA microspheres [27].

In addition to being a regulatory requirement, studies on IVIVC are extremely beneficial as they minimize time, labor costs, and expenses needed to perform human or animal studies with conventional or extended release products. Further, having an IVIVC reduces the need for unnecessary use of human subjects or animals to evaluate drug release [7]. While there is plethora of IVIVC studies published on oral dosage forms, there are fewer reports on this subject with long acting injectables, possibly due to the lack of compendial methods that can be used to assess drug release from extended release injectables. The research presented in this paper has addressed this gap by using a systematic scientific approach to selecting an *in vitro* release method and establishing its suitability to predict *in vivo* behavior. The work presented here also confirms that a suitable *in vitro* method can be used as an indirect measurement of drug availability for complex dosage forms such as PLGA microspheres where formulation factors undergo extensive evaluation prior to selection of a suitable copolymer that can provide a desirable release pattern having clinical relevance.

## 4. Conclusions

A modified dialysis method was selected to characterize *in vitro* behavior of Olanzapine PLGA microspheres prepared using varying molecular weights and copolymer composition. This method was able to accurately discriminate the formulations on the basis of release rates. Rank order of drug release *in vitro* and release profiles were nearly similar to those obtained *in vivo*, either by deconvolution (Nelson-Wagner method) or using the fractional AUC method. An excellent 1 : 1 linear Level A correlation between *in vitro* and *in vivo* release was obtained for all the formulations evaluated, suggesting that the modified dialysis *in vitro* technique was suitable for *in vitro* release assessment of Olanzapine PLGA dosage forms.
